# How to diagnose the 22q11.2 deletion syndrome in patients with schizophrenia: a case report

**DOI:** 10.1186/1744-859X-12-29

**Published:** 2013-09-24

**Authors:** Kazutaka Ohi, Ryota Hashimoto, Hidenaga Yamamori, Yuka Yasuda, Michiko Fujimoto, Noriko Nakatani, Kouzin Kamino, Masatoshi Takeda

**Affiliations:** 1 National Hospital Organization, Yamato Mental-Medical Center, Nara, 639-1042, Japan; 2 Department of Psychiatry, Graduate School of Medicine, Osaka University, Osaka, 565-0871, Japan; 3 Molecular Research Center for Children's Mental Development, United Graduate School of Child Development, Osaka University, Osaka, 565-0871, Japan; 4 Department of Molecular Neuropsychiatry, Graduate School of Medicine, Osaka University, Osaka, 565-0871, Japan

**Keywords:** 22q11.2 deletion syndrome, Schizophrenia, Hypocalcemia, Agitation

## Abstract

The 22q11.2 deletion syndrome is caused by a microdeletion of chromosome 22. One third of all patients with 22q11.2 deletion develop schizophrenia-like symptoms. In general, the prevalence of 22q11.2 deletion in patients with schizophrenia is 1%–2%. The 22q11.2 deletion is one of the major known genetic risk factors for schizophrenia. However, clinical differences in the phenotypes between patients with schizophrenia who are 22q11.2 deletion carriers and those who are not are still unknown. Therefore, it may be difficult to diagnose 22q11.2 deletion in patients with schizophrenia on the basis of clinical symptoms. To date, only two Japanese patients with the deletion have been identified through microdeletion studies of patients with schizophrenia in the Japanese population. Herein, we report the case study of a 48-year-old Japanese woman with 22q11.2 deletion who had a 30-year history of schizophrenia. Based on craniofacial anomalies, unpredictable agitation, hypocalcemia, and brain imaging finding, we suspected the 22q11.2 deletion in clinical populations and diagnosed the deletion using fluorescence *in situ* hybridization analysis. To find common phenotypes in Japanese patients with the deletion who have schizophrenia-like symptoms, we compared phenotypes among three Japanese cases. The common phenotypes were an absence of congenital cardiovascular anomalies and the presence of current findings of low intellectual ability, agitation, and hypocalcemia. We propose that hypocalcemia and agitation in patients with schizophrenia may derive from the 22q11.2 deletion, particularly when these phenotypes are coupled with schizophrenia-like symptoms.

## Background

The 22q11.2 deletion syndrome (Online Mendelian Inheritance in Man (OMIM) 611867, also known as the Velocardiofacial syndrome, OMIM 192430, or DiGeorge syndrome, OMIM 188400) is caused by a microdeletion (1.5–3 Mb) of chromosome 22, with an estimated prevalence of 1 in 4,500 live births [1]. The region of the microdeletion includes approximately 35–60 known genes, and most of the genes affected are expressed in the brain [1]. This disorder is mainly found to have a sporadic occurrence. The phenotypic spectrum of this disorder is highly variable, and patients present at any age. The phenotypes include congenital cardiovascular anomalies (74% of patients), craniofacial anomalies (the majority of patients), palatal anomalies (69%), immunodeficiency (77%), developmental delay or learning disabilities (70%–90%), and hypocalcemia (50%) [2]. The 22q11.2 deletion is usually suspected and then diagnosed in patients who are treated for congenital cardiovascular anomalies coupled with other phenotypes through late adolescence and early adulthood. Alternatively, schizophrenia (OMIM 181500) is a common and complex psychiatric disease with strong genetic components with an estimated heritability of approximately 80% [3,4]. Schizophrenia is characterized by positive symptoms, such as delusions and hallucinations, negative symptoms, such as social withdrawal and blunted affect, and cognitive impairments. In late adolescence and early adulthood, one third of all patients with 22q11.2 deletion develop schizophrenia-like symptoms [5]. In general, the prevalence of 22q11.2 deletion in patients with schizophrenia is 1%–2% [6]; 22q11.2 deletion is therefore one of the major known genetic risk factors for schizophrenia. To the best of our knowledge, three studies have investigated the prevalence of 22q11.2 deletion in Japanese patients with schizophrenia [7-9]. The reported prevalence rates, namely 1/268 (0.37%) [7], 1/300 (0.33%) [8], and 0/575 (0%) [9], in the Japanese population are lower than those in other populations [6]. Thus, the 22q11.2 deletion syndrome in the clinical population of patients with schizophrenia has been diagnosed through research methods, such as deletion studies, but not by the clinical symptoms observed. Therefore, it may be less likely for clinicians to suspect and diagnose the 22q11.2 deletion syndrome in schizophrenia patients based on clinical symptoms alone. In this paper, we report the case study of a Japanese woman with 22q11.2 deletion and a 30-year schizophrenia diagnosis. Based on craniofacial anomalies, hypocalcemia, brain imaging finding, and psychiatric symptoms such as agitation, we suggest that the 22q11.2 deletion syndrome may be suspected and diagnosed in patients.

## Case presentation

**Figure 1:**
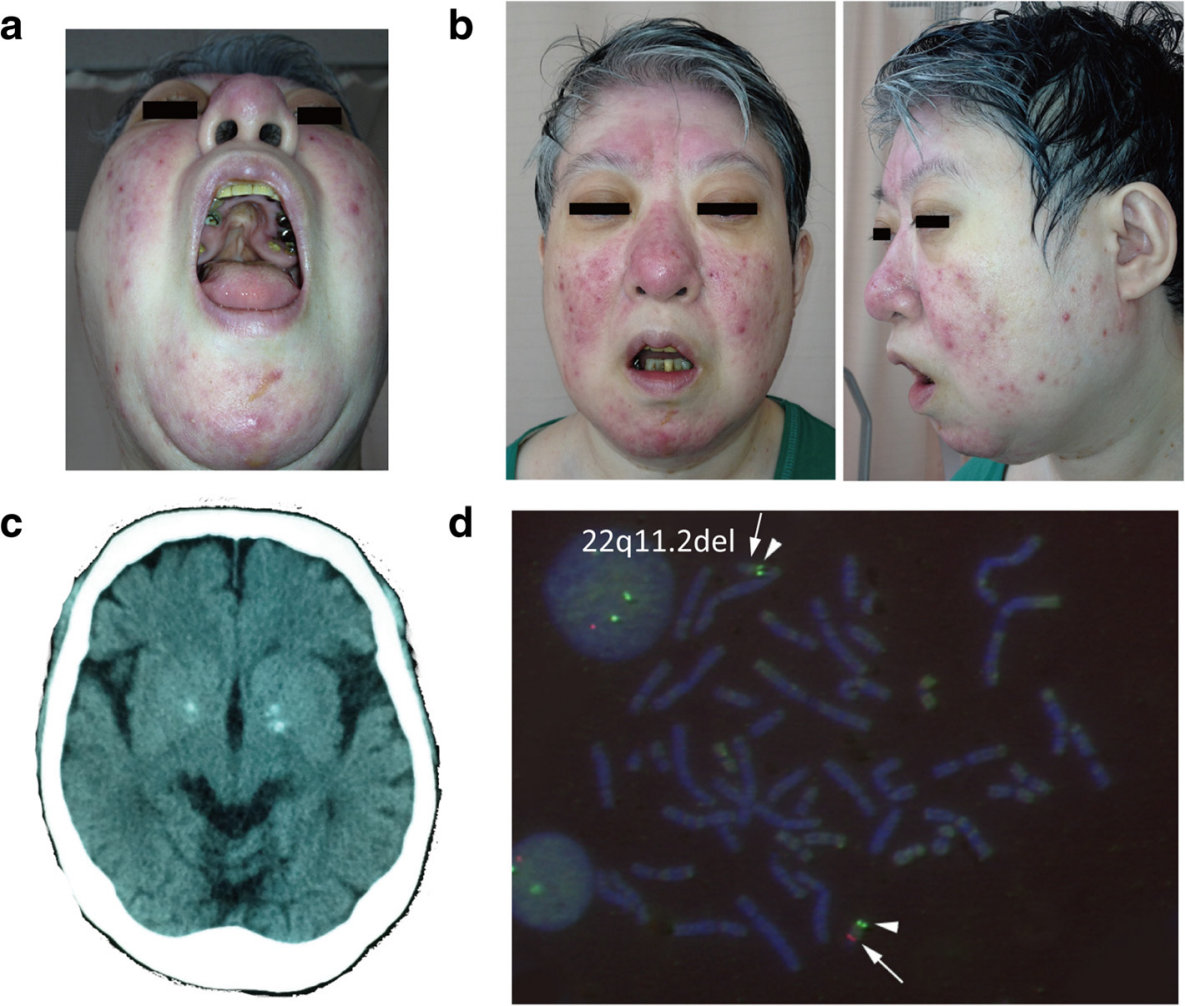
**Features of a 48-year-old woman with the 22q11.2 deletion. (a)** Past history of an operation for cleft palate. **(b)** Mild dysmorphic facial features, including a low anterior hairline, swollen eyelids, malar flatness, nose with a bulbous nasal tip, hypoplastic nasal alae and a square and flat nasal root, small mouth, and a thin upper lip. **(c)** Basal ganglia calcification due to hypocalcemia. **(d)** Fluorescence *in situ* hybridization; *red* and *green* regions were detected by TUPLE1 (22q11.2) and ARSA (22q13.3) probes, respectively. The *arrow* and *arrowhead* indicate expected *red* and *green* regions, respectively. One chromosome 22 with the deletion of TUPLE1 (22q11.2) was detected.

The patient was a 48-year-old Japanese woman who was diagnosed 30 years previously with schizophrenia according to the criteria of the *Diagnostic and Statistical Manual of Mental Disorders, Fourth Edition* (DSM-IV, previously used the *Third Edition*). She was delivered with forceps due to maternal fatigue (birth weight 2,500 g). Her mother had pregnancy-induced hypertension during her pregnancy. No cardiovascular abnormalities had been detected at any routine medical examinations. She had no history of feeding problems, such as regurgitation, nor did she have symptoms of hypocalcemia in early infancy. Her neuromotor development was slightly delayed, with particular delays in the emergence of language. She was a slow learner and finished regular junior high school with the lowest levels of achievement. Since childhood, she was disposed to catching colds, suggesting immunodeficiency resulting from thymic hypoplasia. She had a large cleft palate causing a hypernasal voice and underwent surgery for the cleft palate at 10 years of age (Figure 1a). Her parents and three siblings were alive and well, and no other family members or relatives are clinically affected. Since childhood, the patient had been easily and unpredictably irritated and emotionally labile. At the age of 18, she suffered from auditory hallucinations. Her family was troubled by the unpredictable agitation and behavioral changes associated with the hallucinations and delusions. At the age of 22, she began going to a psychiatric hospital regularly due to her agitation and behavioral changes. She was diagnosed with schizophrenia, and antipsychotic medication was initiated. For transient symptoms associated with hallucinations, delusions, insomnia, and agitation, she was admitted to the hospital at the age of 30. She had been hospitalized for a long time at Yamato Mental-Medical Center. Although she had no history of epilepsy, she experienced a generalized spasm when she had a lumbar vertebral fracture at age 46. At the time of this report, she was 48 years old and was being treated with antipsychotics (risperidone 12 mg/day), with a relatively good control of psychotic symptoms. However, she had persistent irritability, emotional lability, and unpredictable agitation. She underwent surgeries for Basedow disease at 17 years old and for uterine corpus cancer at age 38.

The patient's height was 153 cm, and her weight was 63.5 kg. She had the following typical features of craniofacial anomalies: a low anterior hairline, swollen eyelids, malar flatness, a nose with a bulbous nasal tip, hypoplastic nasal alae and a square and flat nasal root, a small mouth, and a thin upper lip (Figure 1b), as previously reported [10]. Her voice was hypernasal, and she had cavities due to enamel hypoplasia. No abnormality was found on neurological examination. Laboratory examinations revealed hypocalcemia (8.1 mg/dl in serum, normal range 8.7–10.3 mg/dl, serum albumin-corrected calcium level 8.3 mg/dl) and thrombocytopenia (7.2×10^4^ μl^−1^, normal range 13–35×10^4^ μl^−1^). Her intact parathyroid hormone (PTH) level was within normal limits but slightly low (27 pg/ml, normal range 10–65 pg/ml), indicating that the secretion of PTH from the parathyroid was not adequate despite her hypocalcemia. The spectrum of immunodeficiency ranges from absent T cells due to thymic aplasia to normal T cell numbers [11]. In this case, the numbers of CD4+ and CD8+ T lymphocytes were within normal ranges. Brain CT showed bilateral basal ganglia calcification (Figure 1c), which suggests deposition of calcium related to hypocalcemia. Her premorbid intelligence quotient (IQ), assessed by the Japanese Adult Reading Test [12], was 80. To assess current intellectual ability, we used a full-scale IQ of the Japanese version of the Wechsler Adult Intelligence Scale, third edition (WAIS-III) [13]. The WAIS-III did not yield a full-scale IQ (<50; unmeasurable), suggesting that she showed cognitive deterioration. No evidence of cardiovascular disease was found on radiography or electrocardiograph.

The karyotype of this patient was normal (46,XX by G-banding). Fluorescence *in situ* hybridization analysis with the commercially available TUPLE1 (22q11.2) or ARSA (22q13.3) probes indicated that the patient was hemizygous for the TUPLE1 probe (represented by the red region in Figure 1d) and was homozygous for the ARSA probe, represented by the green region. According to the findings, we diagnosed the patient as having 22q11.2 deletion syndrome.

## Discussion

We diagnosed a schizophrenia patient as having 22q11.2 deletion syndrome based on clinical symptoms. To the best of our knowledge, this is the first case in the Japanese population of a patient with schizophrenia-like psychosis to be diagnosed with 22q11.2 deletion syndrome based on clinical symptoms rather than microdeletion studies. The patient presented several typical phenotypes of the 22q11.2 deletion. The disorder is often suspected in the presence of congenital cardiovascular anomalies coupled with other phenotypes in early life. Although her clinical data, such as craniofacial anomalies and cleft palate, were similar to those previously reported in 22q11.2 deletion [2], clinicians could not diagnose her to be with 22q11.2 deletion syndrome based on these phenotypes.

It has been reported that there are no clinical differences in the core phenotype between individuals with schizophrenia who are 22q11.2 deletion carriers and those who are not carriers [14,15]. Psychiatric symptoms and the psychiatric course in patients with the deletion are unremarkable. However, aggression and temper outbursts have been commonly observed in Caucasian patients with the 22q11.2 deletion and schizophrenia-like symptoms [14]. To date, three cases including the present case, which confirmed a diagnosis of the 22q11.2 deletion syndrome following a diagnosis of schizophrenia, have been reported in the Japanese population. Common phenotypes in these cases were an absence of congenital cardiovascular abnormalities and the presence of low intellectual ability (IQ<70), agitation, and hypocalcemia (Table 1). Clinical features prompting a clinician to diagnose a patient with 22q11.2 deletion syndrome vary depending on the age of the patient. Without such classic findings as congenital cardiovascular anomalies, clinicians may miss important diagnostic clues for 22q11.2 deletion in early life. Identification of the 22q11 deletion syndrome, especially in adolescents and adults, may require more careful assessments of their phenotypes apart from congenital cardiovascular anomalies. Current intellectual disabilities are also common in these cases. However, a low IQ is a well-known phenotype in patients with schizophrenia, indicating that it is difficult to distinguish patients with schizophrenia from those with 22q11.2 deletion syndrome on the basis of low IQ. Alternatively, both agitation and hypocalcemia may prompt clinical suspicion and diagnosis of 22q11.2 deletion syndrome in patients with schizophrenia. In the present case, the patient experienced a seizure when she had a lumbar vertebral fracture, which may have resulted in worsening hypocalcemia due to physical stress. In addition, agitation, irritability, and emotional lability in these cases may derive from hypocalcemia. We suggest that an assessment of serum calcium should be added to the routine examination of patients with schizophrenia.

**Table 1:** Common phenotypes of the 22q11.2 deletion syndrome in Japanese patients with a schizophrenia-like presentation

	**Sugama et al. (in 1999) **[7]	**Arinami et al. (in 2001) **[8]	**Ohi et al. (present)**
	**41-year-old woman**	**28-year-old woman**	**48-yearcpaold woman**
Age at onset of psychosis	22	15	18
*Hallucinations and delusions*	+	+	+
*Agitation*	+	+	+
Developmental delay or learning disabilities	+	−	+
*Low current IQ (<70)*	*63*	*61*	*<50*
*Congenital cardiovascular anomalies*	−	−	−
Craniofacial anomalies	+	−	+
Palatal anomalies	−	−	+
Slender tapered figures	NA	+	+
*Hypocalcemia*	+	+	+
Thrombocytopenia	−	−	+
Enamel hypoplasia	NA	NA	+
Basal ganglia calcification	+	NA	+
Immunodeficiency	−	NA	±

Common phenotypes are shown in italics. *NA* not available.

## Conclusions

In this study, 22q11.2 deletion was identified in a 48-year-old woman with schizophrenia-like symptoms as well as hypocalcemia and agitation but without congenital heart malformations. Notably, the absence of congenital cardiovascular anomalies may reduce the probability of identifying 22q11.2 deletion in adult patients with schizophrenia. We suggest that the assessment of serum calcium and agitation, in addition to identification of typical craniofacial anomalies, could aid in the diagnosis of 22q11.2 deletion in adolescent and adult schizophrenia patients. Guidelines for clinicians on diagnosing 22q11.2 deletion in schizophrenia patients are needed.

## Consent

Written informed consent was obtained from the patient and her parents for the publication of this case report and any accompanying images. A copy of the written consent is available for review by the Editor-in-Chief of this journal.

## Abbreviations

DSM: Diagnostic and statistical manual of mental disorders; IQ: Intelligence quotient; OMIM: Online Mendelian inheritance in man; WAIS: Wechsler adult intelligence scale.

## Competing interests

The authors declare that they have no competing interests.

## Authors’ contributions

RH supervised the entire project and was critically involved in the design and interpretation of the data. KO was critically involved in the collection of the data, contributed intellectually to the interpretation of the data, wrote the first draft of the manuscript, and contributed to the editing of the final manuscript. HY, YY, MF, NN, KK, and MT were heavily involved in the collection of data and contributed intellectually to the interpretation of data. All authors read and approved the final manuscript.
